# Optimization of Aflatoxin B_1_-Lysine Analysis for Public Health Exposure Studies

**DOI:** 10.3390/toxins14100672

**Published:** 2022-09-28

**Authors:** Justin B Renaud, Jacob P Walsh, Mark W Sumarah

**Affiliations:** 1 London Research and Development Centre, Agriculture and Agri-Food Canada, London, ON N5V 4T3, Canada; 2 Department of Chemistry, University of Western Ontario, London, ON N6A 3K7, Canada

**Keywords:** AFB_1_-lysine, dried blood spot, volumetric absorptive microsampling, reference serum, biomarker

## Abstract

Aflatoxin B_1_ is a potent human carcinogen produced by several species of *Aspergillus* mainly found on nuts and maize. Exposures in parts of Africa, Latin America and Asia can be at multiples, sometimes orders of magnitude above tolerable daily levels. Although human exposure to aflatoxin can be estimated by analysis of the diet, only determination of the serum albumin aflatoxin adduct provides a health-relevant exposure measure. The lack of a reference serum limits interlaboratory method validation and data comparisons. In this study, we synthetically produced AFB_1_-dialdehyde and covalently coupled it to serum albumin in human serum. This synthetic produced aflatoxin-serum reference material was used in conjunction with isotopically labelled internal standards to evaluate sample digestion methods. This showed using sufficient Pronase in the digestion step was critical to ensure complete proteolytic digestion, which occurs within 4 h. Increasing the digestion temperature from 37 °C to 50 °C also provided a benefit to the overall analysis. In addition, the use of dried blood spots and Volumetric Absorptive Microsampling (VAMS) were investigated showing samples stored with VAMS produced equivalent results to serum samples.

## 1. Introduction

Aflatoxins are carcinogenic mycotoxins produced by *Aspergillus* species. The dominant and most potent of the naturally occurring aflatoxins is aflatoxin B_1_ (AFB_1_) [1,2,3]. Mainly through the consumption of contaminated maize and groundnuts, it is estimated that 500 million people annually in Latin-America, sub-Saharan Africa and Asia are exposed to these carcinogenic hepatotoxins above tolerable levels [3]. Chronic exposure to AFB_1_ causes liver cancer in humans while individuals with concurrent hepatitis B infection are at much greater risk [1,3]. Aflatoxin exposure can also cause additional detrimental effects on women during pregnancy [4]. Our group has previously conducted exposure assessments for aflatoxin, deoxynivalenol, fumonisin and zearalenone to aid hospital-based studies of the health of women and children in Africa [5,6].

The mutagenicity of AFB_1_ arises from phase I metabolic processes of human P450 enzymes specifically, CYP3A4, CYP1A2 and in some individuals, CYP3A5 [7]. These enzymes have the ability to epoxidize the 8,9 vinyl double bond of AFB_1_. AFB_1_-8,9 *exo*-epoxide efficiently chelates between DNA base pairs to react with guanine residues leading to base pair mutations. The AFB1-guanine adduct can be measured in urine [8] and blood [9] as an effective biomarker of acute AFB_1_ exposure. Both endo- and exo-AFB_1_-8,9-epoxide can also undergo rapid hydrolysis to produce AFB_1_-dialdehyde, which forms covalent adducts with human serum albumin (HSA) lysine residues [10,11,12,13,14,15]. The formation of these adducts has been validated as a biomarker of chronic exposure to AFB_1_ [16,17].

Analysis is achieved through protease digestion of HSA to release the AFB_1_-lysine (AFB_1_-Lys) adduct. The resulting AFB_1_-Lys is quantified by LC-MS/MS, HPLC-FLD, or ELISA. Historically, the amount of HSA in the serum has been quantified to normalize the reported AFB_1_-Lys concentration in units of pg/mg albumin. A recent analysis of a large number of samples from many countries suggested that this normalization step may not be necessary [18]. Of the various analytical approaches reported, the most reliable data come from liquid chromatography–tandem mass spectrometry methods [17].

Although the importance of aflatoxin on a global health scale is well understood, there remain critical methodological shortcomings in assessing chronic exposure particularly for disease outcomes other than liver cancer. The 83rd JECFA monograph reported that, “*There remains a continuing need to validate new laboratory methods for aflatoxin–lysine adduct for analytical quality*. *There is also a need for high purity, commercially available, aflatoxin–lysine/aflatoxin–albumin standards for use with LC-MS/MS and other quantitative methods*” [17]. There is an additional barrier to more effective use of biomarker studies namely the lack of alternatives to shipping blood samples over long distances and storage in −80 °C freezers. Recently, progress was made towards addressing the first gap though the publication of a simplified method to synthesize the standards required to quantify AFB_1_-Lys by LC-MS/MS [19].

Studies of exposure to aflatoxin have depended on collecting serum or plasma from affected populations and shipping them on dry ice to labs in the United States, Europe and Canada. The sample collection and shipping portions of this procedure are often cost prohibitive. Alternative and less expensive sampling techniques such as dried blood spots (DBS) have been explored [20]. Another potentially useful technique is Volumetric Absorptive Microsampling (VAMS), which could provide value by providing greater precision of sample volume than DBS.

In this work, the production of an AFB_1_-serum albumin (SA) reference material using blank serum and AFB_1_-epoxide formed via dimethyldioxirane (DMDO) is presented. The reference material was characterized with two different isotopically labelled standards to study the effects of various experimental conditions during sample processing and analysis. Finally, the prepared reference material was used to compare the sample collection techniques of Volumetric Absorptive Microsampling (VAMS) and dried blood spots (DBS) with direct serum analysis.

## 2. Results and Discussion

### 2.1. Coupling of AFB_1_-Dialdehyde to Serum Albumin

Phase I epoxidation of AFB_1_ by CYP1A2 produces both the *exo*- and *endo*-AFB_1_-8,9-epoxide diastereoisomers, while CYP3A4 only produces the *exo*-epoxide form [21]. DMDO is an efficient epoxidizing reagent for AFB_1_ and produces largely the *exo*- isomer [22] with only minor amounts of the *endo*- [23]. Although other epoxidation reagents such as *m*-chloroperoxybenzoic acid have also been used to generate AFB_1_-8,9-epoxide and is a more stable epoxidation reagent than DMDO, which has a limited shelf-life, it produces approximately 22% of the unwanted *endo* stereoisomer [24]. In this work DMDO was used to produce the desirable *exo*-AFB_1_-8,9-epoxide. AFB_1_-epoxide undergoes rapid hydrolysis in an aqueous environment to form the AFB_1_-dihydrodiol, which is in equilibrium with AFB_1_-dialdehyde [12]. The initial AFB_1_ solution in dichloromethane (DCM) was clear, however following the addition of DMDO, drying and reconstitution in 0.1 M sodium bicarbonate (pH 8.1), the solution was an intense yellow colour, in line with previous observations that indicated the presence of the dialdehyde [19]. To maximize the amount of AFB_1_ adducts on the reference material (RM) serum, while ensuring that the adducts formed were similar to those formed in vivo it was necessary to determine the theoretical maximum molar ratio of AFB_1_ to HSA. Based on previous experiments performed on bovine serum albumin by Guengerich (2002) [10], it is believed that up to two lysine residues (Lys_455_, and Lys_548_) will react with AFB_1_-dialdehyde in vivo. Both of these bovine serum albumin Lys residues have homologous residues in HSA, Lys_456_ and Lys_549_, respectively. This theoretical maximum of two AFB_1_ adducts per HSA protein, 100 μg of AFB_1_ (320 nmol) was converted to AFB_1_-dialdehyde and reacted with 900 μL of HSA (31.4 mg HSA/mL serum; 425 nmol). Assuming that the epoxidation of AFB_1_ and its subsequent aqueous hydrolysis was quantitative, this represents a maximum molar ratio of 0.75 mol AFB_1_/mol_HSA_. This ratio would minimize the potential for additional AFB_1_-adduct formation that would not occur in vivo. Following the incubation of AFB_1_-dialdehyde with the human serum, the serum became yellow after extensive dialysis. This suggests that the AFB_1_-dialdehyde chromophore was covalently attached to protein.

### 2.2. Factors Affecting Pronase Digestion

The synthetically produced AFB_1_-HSA serum allowed for the careful investigation of experimental conditions that have previously been employed in this analysis. To date, the main technique used to release AFB_1_-Lys from HSA is through extensive proteolysis by Pronase in PBS buffer, however a more detailed examination of the digestion conditions is warranted. Specifically, a better idea that complete proteolysis and release of AFB_1_-Lys is occurring in previously published methods to ensure that the results published to date represent the true adduct concentrations. In 2005, McCoy et al. examined the effects of digestion time with Pronase concentration on the appearance of the liberated AFB_1_-Lys [25]. Typically, Pronase digestion times in the literature vary between 12–21 h, [26]. However, McCoy showed that relatively high ratios of protein: Pronase (approx. 5:1), resulted in maximum release of AFB_1_-lysine by 4 h incubation at 37 °C. In addition, the authors reported that the rate of digestion was reduced at lower Pronase concentrations but not the overall extent of hydrolysis.

**Figure 1:**
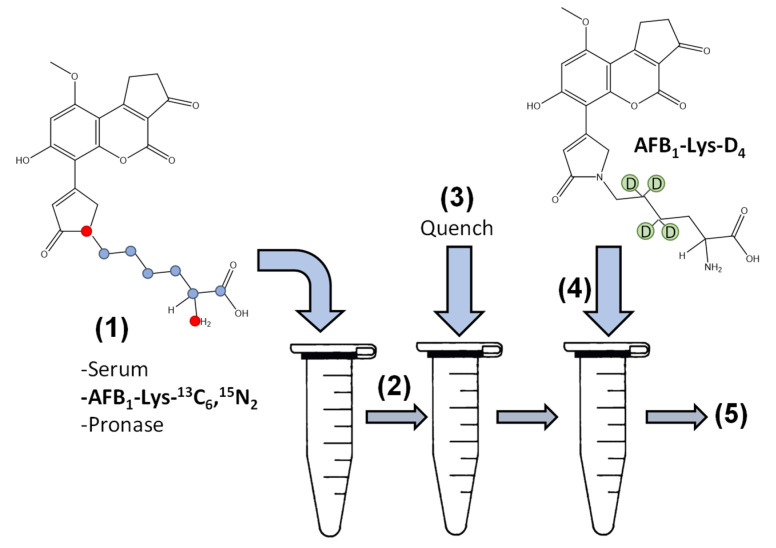
Two isotopically labelled internal standards were used to optimize and evaluate the analytical method. (1) Serum containing the AFB_1_-adduct was added with buffer and the AFB_1_-Lys-^13^C_6_,^15^N_2_ internal standard followed by Pronase. (2) The Pronase containing mixture was incubated to allow proteolysis and (3) quenched by the addition of methanol. After quenching, (4) the second internal standard, AFB_1_-Lys-D_4_ was added and the mixture and (5) analyzed by LC-MS/MS.

To explore this an initial digestion experiment was performed by adding Pronase to serum protein ratios of 1:2, 1:5 and 1:10 over 24 h. The use of two different isotopically labelled internal standards allows for the determination of signal suppression and enhancement (SSE%), the stability of the internal standards during the digestion and the release of AFB_1_-Lys from the serum albumin to be monitored independently throughout the incubation (Figure 1).

**Figure 2:**
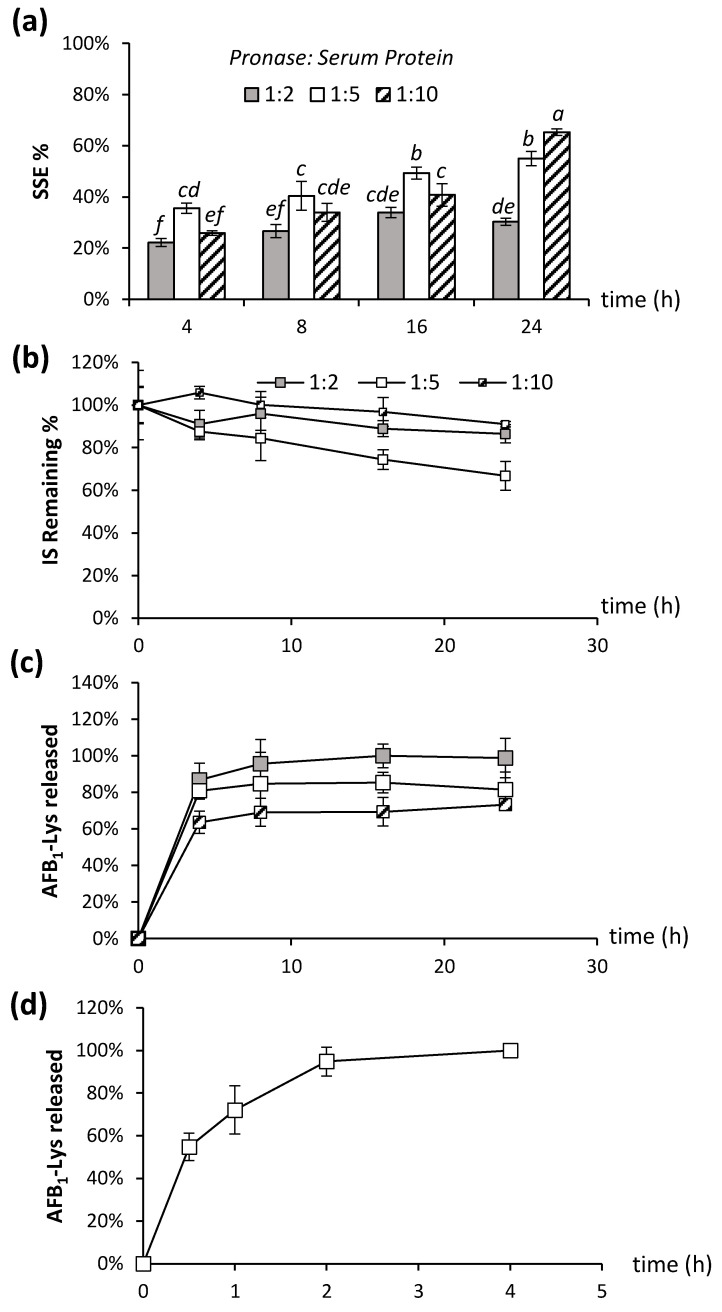
The amount of Pronase used and incubation time was examined in terms of effect on (**a**) SSE%, (**b**) Stability of the internal standard (AFB_1_-Lys-^13^C_6_,^15^N_2_) (**c**) AFB_1_-Lys adduct released over 24 h and (**d**) AFB_1_-Lys adduct released over 4 h. SSE% was determined using the peak area of AFB_1_-Lys-D_4_ added after incubation. The stability of an internal standard was determined using the peak area of AFB_1_-Lys-^13^C_6_,^15^N_2_, which is added prior to incubation. AFB_1_-SA adduct released was determined using peak area of AFB_1_-Lys corrected for SSE%. Pronase: Serum protein ratios were 1:2, 1:5 and 1:10. The SSE% resulting from different digestion conditions and denaturants bearing different letters are significantly different (Tukey’s test, *p* < 0.05).

SSE% was determined as the ratio of peak area of AFB_1_-Lys-D_4_ added after the incubation is quenched to the peak area of AFB_1_-Lys-D_4_ in a solution containing only buffer. The stability of the internal standard during the digestion could then be monitored using the peak area of AFB_1_-Lys-^13^C_6_,^15^N_2_ added prior to the addition of Pronase and corrected for SSE% by the 2nd internal standard AFB_1_-Lys-D_4_ (Figure 2).

Signal suppression was made worse by increasing the concentration of Pronase; at 24 h, the SSE% was 1.8 times higher in the 1:5 Pronase: serum mixture compared to the 1:2 (*t*-test, *p* = 0.003) (Figure 2a). Conversely, SSE% improved as the incubation time increased from 4 to 24 h for all tested conditions (Tukey’s test, *p* < 0.05). This initial result suggests that a more complete digestion of the proteins into individual amino acids improved the signal suppression, likely as these individual amino acids and/or small peptides are not retained during the online SPE step, and thus will not co-elute with the target analyte. Previous analytical methods for AFB_1_-Lys have only employed a single, isotopically labelled internal standard added prior to the liberation of the target analyte via proteolysis. This means that it is possible that the internal standard may not experience the same reaction conditions as endogenous AFB_1_-Lys, which will be released from the intact protein overtime. After 4 h of incubation, the AFB_1_-Lys-^13^C_6_,^15^N_2_ internal standard was not significantly degraded (*t*-test, *p* > 0.05) in any of the Pronase: protein ratios compared to the beginning of the incubation. However, at time points of 8 h, 16 h and 24 h, there was significant degradation (*p* = 0.02, *p* = 0.025 and *p* = 0.01, respectively) in the 1:5 Pronase:Serum protein ratios (Figure 2b). At the lowest Pronase concentration, no significant degradation of the internal standard was observed after 24 h (Figure 2b; *t*-test, *p* > 0.05).

Finally, the digestion efficiency of the various Pronase ratios over time was evaluated. AFB_1_-Lys released from the intact serum albumin was monitored and corrected for SSE% using the AFB_1_-Lys-D_4_ internal standard (Figure 2c). In agreement with McCoy et al., (2005) [25], we observed that a 4 h incubation at Pronase: protein ratios 1:5 and 1:2 resulted in extensive release of the AFB_1_-SA adduct as AFB_1_-Lys. However, we observed that the total AFB_1_-SA released was not complete at lower Pronase concentrations, even after extended digestion times (Figure 2c). In fact, there was no significant (*t*-test, *p* > 0.05) difference in the amount of AFB_1_-Lys released between 4 h and 24 h incubation for any of the Pronase ratios tested. As the majority of AFB_1_-Lys released appears to occur during 4 h of incubation, the incubation was repeated at shorter intervals of 0, 30 min, 1 h, 2 h, and 4 h using the previously established protein: Pronase ratio 5:1 (Figure 2d). In this experiment, normalized to the maximum value of AFB_1_-Lys, it is clear that the reaction is very rapid, occurring almost to completion after just two hours. Taken together, these data suggest that insufficient Pronase concentration will result in an underestimate of AFB_1_-Lys, more extensive digestion leads to improved SSE%, and increased digestion times above 4 h can lead to significant degradation of the internal standard as well as endogenous AFB_1_-Lys.

### 2.3. Effects of Buffers and Denaturants on Digestion

**Figure 3:**
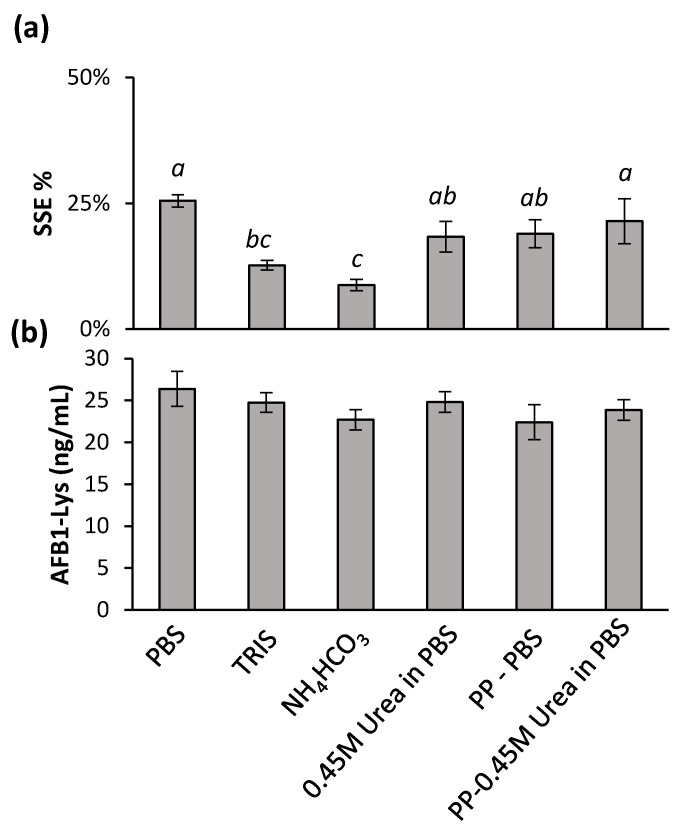
The effects of digestion buffer and a urea denaturant on (**a**) SSE% relative to a standard in 50% MeOH and (**b**) AFB_1_-SA release was examined. Results were subjected to single-factor ANOVA and Tukey’s multiple comparison of means. The SSE% resulting from different digestion conditions and denaturants bearing different letters are significantly different (Tukey’s test, *p* < 0.05). The conditions tested had no significant effect on the AFB_1_-Lys released (Tukey’s test, *p* > 0.05).

To address the issue of significant SSE% and possible approaches to enhance proteolysis while decreasing the digestion time and/or amount of Pronase used, a series of buffers and denaturing techniques were investigated. A Pronase:protein ratio of 1:5 was used and digestion was quenched after 4 h. Buffers of PBS, TRIS, and a mass spec compatible 50 mM ammonium bicarbonate were used during the digestion (Figure 3).

All three buffers used during the digestion did not result in any improvement in digestion efficiency, however both TRIS and ammonium bicarbonate decrease SSE%. In an effort to improve the digestion efficiency, a number of denaturant methods were examined. First, the enriched serum was incubated with 4 M Urea for 30 min and diluted to a final concentration of 0.45 M prior to the addition of Pronase. Similarly, prior to digestion, the serum proteins were precipitated by the addition of methanol, the supernatants discarded and reconstituted in either PBS buffer or buffer with 0.45 M Urea. Compared to PBS alone, there was a minimal, yet significant (*t*-test, *p* < 0.05) decrease in SSE with a protein precipitation step, or the addition of urea separately, however no significant difference was observed with a protein precipitation and added urea together (Figure 3a). This confirmed that a protein precipitation step, such as that used for serum metabolomics analysis is compatible with a separate serum-adduct analysis from the pellet [5,27]. HSA contains 17 disulfide bonds [28], and therefore, dithiothreitol was also explored as a reducing agent to assist in denaturing the proteins and increasing digestibility. However, a complete disappearance of both the released AFB_1_-Lys and internal standards was observed showing the use of DTT to be incompatible with this analysis (data not shown).

### 2.4. Effects of Digestion Temperature

**Figure 4:**
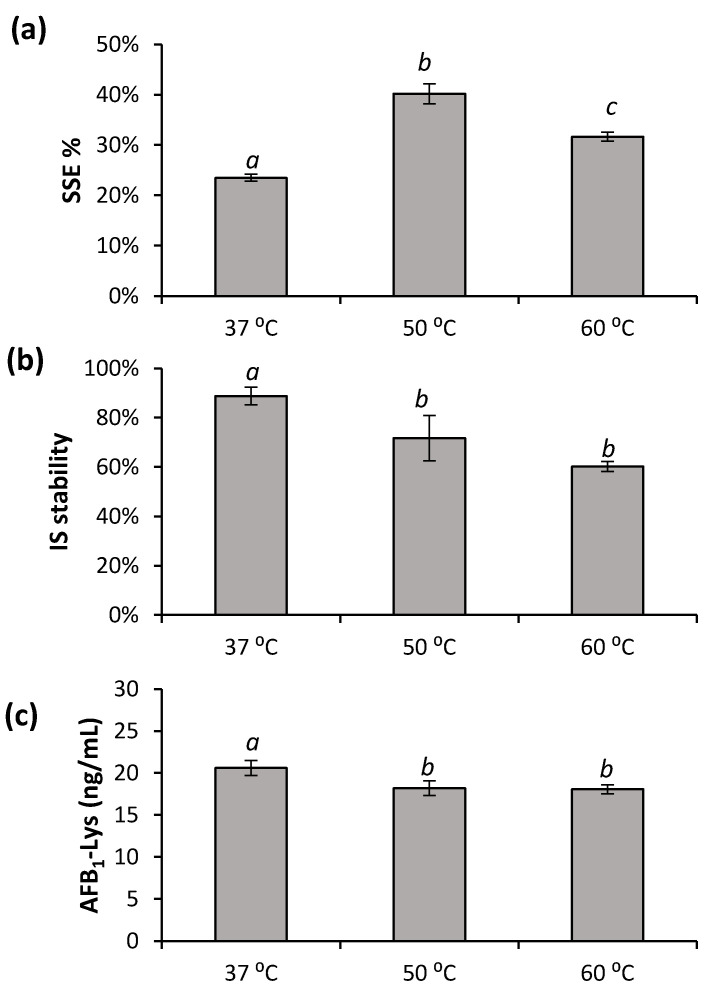
The effects incubation temperature (**a**) SSE%, (**b**) stability of AFB_1_-Lys-^13^C_6_,^15^N_2_ internal standard (IS) and (**c**) AFB_1_-SA adduct released was examined. The results arising from different digestion temperatures bearing different letters are significantly different (Tukey’s test, *p* < 0.05). Over the 4 h incubation, each temperature had a significant effect on (Tukey’s test, *p* < 0.05) SSE%; 50 °C resulted in the best SSE%. The degradation of internal standard was also increased by temperature. A minimal, yet significant (*t*-test, *p* < 0.05) increase in AFB_1_-SA adduct released was observed at 37 °C while no difference was observed between 50 °C and 60 °C.

Pronase is effective above room temperature. Higher temperatures might contribute to HSA denaturation and/or improved digestion. However, the effects of digestion temperature on the release of AFB_1_-Lys has not been reported. The effects of digestion temperatures of 37 °C, 50 °C and 60 °C on SSE%, internal standard stability and digestion efficiency are shown in Figure 4.

An elevated temperature of 50 °C showed a significant improvement in SSE% compared to the traditional 37 °C and a high temperature of 60 °C (Figure 4a). However, this came at the expense of a reduction in the stability of the internal standard that was added prior to the digestion (Figure 4b). At 37 °C, 20.6 ± 0.9 ng/mL of AFB_1_-Lys was released during digestion compared to 18.1 ± 0.9 ng/mL and 18.0 ± 0.5 ng/mL for 50 °C and 60 °C, respectively. Although the values are similar, the AFB_1_-Lys released is significantly more than the higher temperatures (Figure 4c; Tukey’s test, *p* < 0.05). Similar to what was found with different Pronase ratios (Figure 2), after a point, increasing the total digestion of HSA does not improve the amount of AFB_1_-Lys released. However, as more peptide fragments were further digested into smaller peptides and individual amino acids, the signal suppression improved. Previously, AFB_1_-Lys has been enriched from the serum digestate using a mixed-mode Oasis^®^ Max SPE cartridge (Waters, Milford, USA). To improve any downstream enrichment, or allow for the use of other techniques such as dilute-and-shoot or online-SPE it is important to improve the SSE% at the digestion step as much as possible.

### 2.5. Combined Effects of Temperature and Pronase Concentration on Method Performance

**Figure 5:**
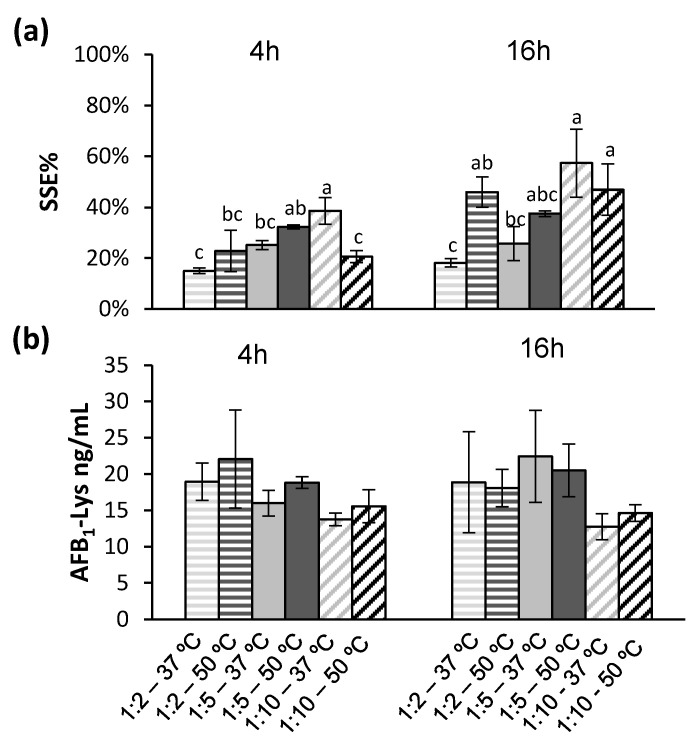
Based on the observation that increased temperature led to an improved SSE% while increased concentration of Pronase will decrease SSE%, several combinations were examined. The SSE% resulting from different digestion conditions bearing the same letter are not significantly different by Tukey’s (*p* < 0.05); 4 h and 16 h incubations were compared separately. (**a**) The SSE% was significantly (Tukey’s test, *p* < 0.05) improved by increasing temperature. (**b**) Although regardless of temperature and time, a lower concentration of Pronase resulted in incomplete digestion and liberation of the AFB_1_-Lys adduct, these results were not significantly different from each other (Tukey’s test, *p* > 0.05).

Based on the findings that an increase in overall protein digestion leads to significant improvements in signal suppression, while increasing the amount of Pronase will also worsen SSE%, a series of Pronase ratios, and incubation temperatures were compared after 4 h and 16 h (Figure 5).

Increasing the incubation temperature of the 1:5 Pronase: protein ratio improved the SSE% (Figure 5a). Even with increasing the reaction temperature to 50 °C and a 16 h incubation time, a Pronase: protein ratio of 1:10 resulted in incomplete release of AFB_1_-Lys (Figure 5b). Unlike the previous experiment examining temperature at only a 1:5 Pronase: protein ratio over 4 h (Figure 4c), no temperature had no significant effect (Tukey’s test, *p* > 0.05) in any of the Pronase: protein ratios tested (Figure 5b).

In summary, a 1:5 Pronase: protein ratio with an incubation time of 4 h and an increased temperature of 50 °C will minimize SSE% and maximize the AFB_1_-Lys released. In addition, the 4 h incubation period minimizes the degradation of internal standard and endogenous analyte. This examination of the digestion conditions showed that the conditions that have been previously used, namely the digestion time, buffer, and amount of Pronase [25] did result in the maximum release of the AFB_1_-Lys adduct, adding confidence to previously reported values.

### 2.6. Comparison of Sample Collection Techniques

As noted, AFB_1_-SA adduct is typically determined directly from a serum or plasma sample. As an alternative, Dried Blood Spots have shown promise since measured values were normalized by the amount of HSA, not serum volume [20]. There is recent evidence that suggests that aflatoxin serum adducts should be normalized by serum volume and not by HSA [18]. This means that unless the volume of serum or blood is carefully applied to a DBS card prior to desiccation and shipping, it may not easily compatible with this analysis. One alternative device, VAMS, is a microsampling technique where the volume of collected material is carefully controlled [29]. However, to our knowledge they have not yet been examined for the analysis of HSA bound contaminants. VAMS has a maximum sample collection volume threshold, whereas DBS do not. A drawback however is that although it is possible to collect more samples with DBS by simply increased the size of the spot, the sample collection volume of VAMS is limited to what is commercially available from the vendor (currently a maximum of 30 μL).

A critical advantage of synthesizing RM of serum with a high concentration of the AFB_1_-adduct, is that it can be blended with blank serum to produce various concentrations and determine if methods will have a concentration based bias. The RM AFB_1_-serum produced in this work was blended with blank serum to generate material that was at approximately 0.1×, 0.5× and 2× the concentrations used in the assays above. 20 μL of this material was either spotted onto a Whatman 903 DBS filter paper (Millipore Sigma, Burlington, MA, USA), or onto a 20 μL VAMS sampler device (Neoteryx™,Torrance, CA, USA). Similarly, 20 μL of direct serum was also analyzed. All samples were digested at a 1:5 Pronase: protein ratio, at 50 °C.

**Figure 6:**
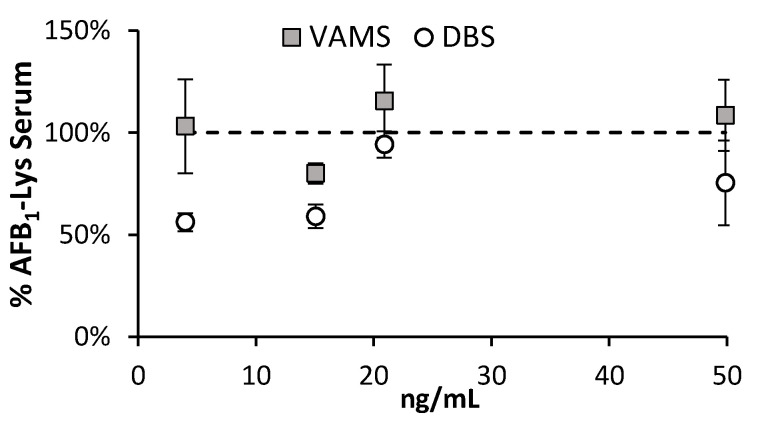
The measured concentrations of liberated AFB_1_-SA were compared for samples collected using DBS and VAMS systems. In comparison, the VAMS systems showed high similarities with the SSE% and measured concentrations produced by direct serum sample.

The average peak area of the AFB_1_-Lys-D_4_ internal standard added post-digestion showed that there was no difference between serum that was directly processed, and serum within the VAMS device. There was a significant increased signal suppression in samples collected via DBS (Figure 6), however this could have resulted from the direct digestion of the protein on the DBS as no initial extraction step was used. Owing to the generation of characterized RM material, the concentration of AFB_1_-SA can be controlled through blending the enriched material with blank serum. Doing so, there is good agreement between the AFB_1_-Lys concentration measured in VAMS collected material, with serum directly.

## 3. Conclusions

In this study we characterized an AFB_1_-Lys adduct serum albumin reference material for method validation and data quality assurance. Additionally, we re-examined the historically used sample preparation steps and showed that VAMS is a promising technique for AFB_1_-Lys analysis but suggested that 20 μL samplers are too small to be useful. Field work would require VAMS with a capacity of ≥100 μL. Future work will involve the use of the reference material for an interlaboratory method comparison and validation study. These methods will also be applied to human populations for the determination of AFB_1_-Lys.

## 4. Materials and Methods

### 4.1. Materials

AFB_1_ was obtained from Toronto Research Chemicals (Toronto, ON, Canada). The LC-MS grade solvents H_2_O, methanol, acetone and acetonitrile were purchased from Fisher Scientific (Ottawa, ON, Canada). DCM (anhydrous) was purchased from Fisher Scientific (Ottawa, ON, Canada). Blank serum (H4522; from human plasma, USA origin, sterile-filtered; Millipore Sigma Burlington, MA, USA), sodium bicarbonate and Oxone^®^ was purchased from Sigma-Aldrich (Oakville, ON, Canada). Labelled AFB_1_-Lys adduct was prepared according to Renaud et al., (2022) [19]. In that work, which minimized the production of unwanted reaction by-products, quantitative NMR was used to demonstrate that the previously reported molar attenuation coefficient (ε_400_ 30,866/M cm) [30] used for the generation of AFB_1_-Lys standards is valid.

### 4.2. Synthesis of Fortified AFB_1_-HSA in Human Serum

AFB_1_ was solubilized in acetone at a concentration of 1 mg/mL. The solution was sonicated briefly to ensure that the solid residue was completely dissolved. 100 μL of this solution, representing 100 μg of material was transferred to a 2 mL amber glass vial and dried at 45 °C using a hot plate. After drying, 200 μL of anhydrous DCM was added and the solution was again dried. 200 μL of anhydrous DCM was added and a solution of DMDO was added in a molar ratio of 2.5 DMDO to 1 AFB_1_. The production of DMDO using Oxone^®^ and acetone is described elsewhere [5]; the concentration was determined by UV-Vis [31]. The sample was incubated at room temperature for 4 h. 100 μL of 0.1 M sodium bicarbonate (pH 8.1) was added to the solution and the vial was placed at 45 °C until all the DCM had evaporated. The presence of the AFB_1_-dialdehyde could be observed by an intense yellow colour in the solution [19]. 900 μL of sterile-filtered human serum was added to AFB_1_-dialdehyde solution and vortexed for 15 s. The serum solution was then incubated with gentle shaking for 16 h overnight at room temperature. To remove un-coupled aflatoxin and AFB_1_-dialdehyde, the serum was transferred to a Pierce 3 mL 3.5 kDa Dialysis cassette (Thermo Scientific, Waltham, MA, USA) and exchanged in 500 mL of PBS solution (pH 7.2) overnight. The PBS solution was removed and replaced with 500 mL of fresh buffer and exchanged for 10 h. Finally, the buffer was replaced with fresh PBS and exchanged overnight again. The dialyzed serum was removed from the cassette and freeze dried. The serum residue was finally reconstituted in 1 mL of LC-MS grade H_2_O where its colour was noticeably more yellow than prior to the addition of the AFB_1_-dialdehyde.

### 4.3. Digestion Assay Conditions

First, 10 μL of fortified serum was diluted into 90 μL of PBS buffer. 59 μL of this 10× diluted solution was spiked into 941 μL of blank human serum. This enriched serum was subsequently used for all reaction assays (Figure 1).

25 μL of serum was added to a 1.7 mL microcentrifuge tube on ice, followed by 8.5 μL of AFB_1_-Lys-^13^C_6_,^15^N_2_ internal standard (25 ng/mL). 111.5 μL of buffer (PBS, PBS with 0.45 M Urea, TRIS or ammonium bicarbonate all adjusted to pH 7.5), water and finally Pronase was added. The amount of water was adjusted based on assay conditions so that the final volume was 200 μL in all experiments. The mixture was incubated on a F1.5 thermomixer (Eppendorf, Mississauga, ON, Canada) at 700 rpm at either 37 °C, 50 °C or 60 °C. After incubation, the reaction was quenched by the addition of 200 μL of methanol. 8.5 μL of the second internal standard, AFB_1_-Lys-D_4_ (25 ng/mL) was then added, the samples were vortexed briefly and centrifuged at 8000 rpm for 10 min at 4 °C. 200 μL of supernatant was transferred to a polypropylene HPLC vial for LC-MS/MS analysis. For assays that performed a protein precipitation step prior to digestion, 200 μL of methanol was added to 25 μL of serum, which was vortexed and centrifuged at 8000 rpm for 10 min at 4 °C. The supernatant was discarded and the pellet was resolublized with PBS buffer or PBS with urea as described above.

### 4.4. Volumetric Absorptive Microsampling and Dried Blood Spots

20 μL Volumetric Absorptive Microsampling (VAMS) devices were obtained from neoteryx™ (Torrance, CA, USA), and Whatman^®^ 903 protein saver cards were obtained by Millipore Sigma (Burlington, MA, USA). For both DBS and VAMS 20 μL of enriched serum was placed by pipetted onto the support. The devices were allowed to air dry for 60 min, afterwards, 8.5 μL of internal standard of AFB_1_-Lys-^13^C_6_,^15^N_2_ was also placed via pipette onto the supports and allowed to air dry for an additional 15 min. The VAMS device was removed and placed into a 2 mL polypropylene microcentrifuge tube. The DBS paper was carefully excised and cut into 8 pieces with a scalpel, which were placed in a 2 mL polypropylene microcentrifuge tube. 111.5 μL of PBS buffer (pH 7.5) and 40 μL of H_2_O were added followed by 20 μL of Pronase (17.5 mg/mL). Samples were incubated at 50 °C for 4 h, supernatants were removed and 200 μL of methanol added to quench the reaction. 8.5 μL of the second internal standard, AFB_1_-Lys-D_4_ was added and 200 μL was transferred to a 250 μL polypropylene HPLC vial.

### 4.5. Online SPE—LC-MS/MS

Processed samples were analyzed by a Thermo Vanquish™ Duo, tandem UHPLC system coupled to TSQ Altis™, triple quadrupole mass spectrometer (Thermo Fisher Scientific, Waltham, MA, USA). Sample vials were stored in an autosampler at 10 °C. 100 μL of each sample was injected onto a 2 cm Thermo Aq online SPE column (Thermo Fisher Scientific, Waltham, MA, USA) using Mobile phase A (H_2_O + 0.1% formic acid (FA); Optima LC-MS Grade) at a flow rate of 600 μL min^−1^ for 3.5 min. Following the injection, the trapped analytes were eluted off the online-SPE column and onto an analytical, Agilent Zorbax Eclipse Plus; (2.1 × 50 mm, 1.8 μm; Mississauga, Canada); maintained at 35 °C with a flow rate of 300 μL min^−1^. Mobile phase B (acetonitrile + 0.1% FA; Optima LC-MS Grade) was increased from 2% to 100% over 3 min held for 1 min. Mobile phase B was returned to 2% over 30 s and held for 1 min prior to the next injection. The OptaMax NG H-ESI source (Thermo Fisher Scientific, Waltham, MA, USA) was operated with capillary voltages of 3.5 kV in positive ionization mode, ion transfer tube temperature of 325 °C and vaporizer temperature of 350 °C. The sheath, auxiliary and sweep gases were set to 25, 10 and 1 arbitrary units, respectively. Target analytes and their corresponding internal standards were monitored using the settings listed in Table 1. Quantification was performed in Xcalibur^®^ Quan Browser (Thermo Fisher Scientific, Waltham, MA, USA). Statistical analysis were performed in R, using either a *t*-test (*p* < 0.05) to compare two measurements or single-factor ANOVA and Tukey’s multiple comparison of means (*p* < 0.05) to compare more than two measurements.

## Figures and Tables

**Table 1:** LC-MS/MS analyte details.

Analyte	Ion Type	RT (min)	Precursor *m/z*	Quantifier *m/z* (CE)	Qualifier *m/z* (CE)
AFB_1_-Lys	[M + H]^+^	2.41	457.2	394 (25)	411 (19)
AFB_1_-Lys (^13^C_6,_^15^N_2_)	[M + H]^+^	2.41	465.2	400 (25)	418 (19)
AFB_1_-Lys (D_4_)	[M + H]^+^	2.41	461.2	398 (25)	415 (19)

## Data Availability

Not applicable.
